# Wellbeing and Resilience in Tourism: A Systematic Literature Review During COVID-19

**DOI:** 10.3389/fpsyg.2021.748947

**Published:** 2022-01-05

**Authors:** Margarida Pocinho, Soraia Garcês, Saúl Neves de Jesus

**Affiliations:** ^1^CIERL, University of Madeira, Funchal, Portugal; ^2^Research Centre for Tourism, Sustainability and Well-Being (CinTurs), University of Algarve, Faro, Portugal

**Keywords:** wellbeing, resilience, COVID-19, tourism, positive psychology

## Abstract

The United Nations World Tourism Organization (UWTO) has acknowledged 2020 as the worst year in tourism history due to the worldwide pandemic COVID-19. Destinations, tourists, local communities, stakeholders, and residents, and their daily activities were affected. Thus, wellbeing and resilience are two crucial variables to help the industry and the people recover. This research aims to analyze early positive approaches and attitudes to respond to the negative impact of COVID-19 in tourism everyday activities that have at its core wellbeing and resilience, the two main variables of the Positive Psychology field of studies. A systematic literature review was conducted, following PRISMA guidelines to achieve this aim. The research was done using the Online Knowledge Library (B-on) and all the available databases. The research led to 32 articles that were screened using the inclusion and exclusion criteria. A total of 18 scientific articles met all criteria. Overall, results show that a positive and resilient approach to deal with the adverse outcomes of the pandemic is a concern for stakeholders and the future of the organizations in the tourism and hospitality sector, as is tourists’ wellbeing. However, less research has been done on wellbeing and a clear lack of research regarding residents’ wellbeing and resilience is evident. A deeper study of wellbeing and resilience in tourism is needed, and actual practices and interventions to ensure that all tourism actors have the resources to overcome the pandemic and restart the industry’s daily lives feeling well and safe.

## Introduction

In 2015, the United Nations launched the 2030 agenda for Sustainable Development with 17 goals to transform the world. The overall aim of this agenda was (and it still is) to promote a more peaceful, resilient, and equitable world while keeping in mind the sustainability of the planet (The Lancet Public Health, 2020). The array of Sustainable Development Goals (SDGs) includes health and wellbeing as one of these and assumes it as a priority for all ages (The Lancet Public Health, 2020).

Wellbeing can be seen as a practice or a process related to living a good life (Buzinde, 2020). The study of wellbeing has in Positive Psychology one of its main streams since this field is “(…) the scientific study of the strengths, characteristics, and actions that enable individuals and communities to thrive” (Seligman, 2013, p. 2).

Tourism can be a direct or indirect contributor to all sustainable goals (Santos et al., 2020), including wellbeing. Scholars have considered Positive Psychology and subsequently, the study of wellbeing in tourism a natural step in the field that can support product innovation, the tourism experience, and leads to the competitiveness of tourism (Garcês et al., 2020). Tourism has three base and important actors: tourists, destinations/locals, and stakeholders/workers. A balance between these is crucial to ensure the continuous improvement of the industry because one cannot exist without the other (Garcês et al., 2020). Tourism experiences can improve the wellbeing of residents and tourists, and wellbeing can be a creative opportunity to innovate in destinations (Garcês et al., 2018). However, studies in this field have focused mainly on tourists, with a noticeable lack of research about positive psychology variables focused on local communities and tourism workers (Vada et al., 2020). Nevertheless, research has shown that tourists’ wellbeing is influenced by relationships, learning of a new place and culture, and/or learning new skills. Thus, initiatives that involve tourists within the community, such as volunteer activities, will promote tourists’ wellbeing, but not only locals, the community and even the place sustainability can gain from these experiences (Vada et al., 2020).

However, COVID-19 led tourism activities to an unprecedented loss worldwide. From January 2020 to March 2021, there were 180 million fewer arrivals worldwide (UNWTO, 2021b). The lowest numbers were seen in Asia and the Pacific, followed by Europe, Africa, Middle East, and the Americas (UNWTO, 2021b). In January 2021, the number of international tourists’ arrivals was 87% less than in January 2020 (UNWTO, 2021c). In February 2021, 32% of worldwide destinations were entirely shut down to international arrivals, 34% partly closed, and only 2% have relaxed travel restrictions (UNWTO, 2021a). From an economic perspective, the pandemic led to a drop of 64% in receipts (UNWTO, 2021b). Destinations, residents, and tourists were (and still are) affected by the travel restrictions. While hope for improvement exists, particularly with the vaccination, experts believe that achieving 2019 numbers will only be possible after 2024 and maybe later (UNWTO, 2021b).

The pandemic is a threat to progress made in the sustainable development goal tree that looks to ensure health and wellbeing for all (The Lancet Public Health, 2020). It has had severe consequences in society, the environment, and in people’s health and wellbeing (Passavanti et al., 2021). Pandemics and other health crises lead to a growth in mental health problems, influencing tourists’ behaviors, and also their wellbeing (Abbas et al., 2021). However, COVID-19 will also impact the well-being of those who work in tourism (McCartney et al., 2021). Unemployment, panic generated by COVID-19, and lack of social support are considered key hazards to the tourism and hospitality employees’ perceived wellbeing (Chen, 2020). In already done studies, in the context of COVID-19, job insecurity has had a significant effect on hotel employees’ anxiety and depression, and resilience was a moderator reducing the negative impact of job insecurity in depression (Aguiar-Quintana et al., 2021). Overall, research shows that COVID-19 pandemic–perceived risk produces uncertainty and fear, leading to increased stress and vulnerability, and subsequently to a loss of mental wellbeing (Paredes et al., 2021). Threat severity and susceptibility can trigger fear of traveling, yet it can also lead to protective travel behaviors. The fear of traveling can induce coping strategies, increasing individuals’ resilience, and embracing careful travel behaviors (Zheng et al., 2021).

With the ongoing pandemic tourists, behavior patterns are expected to change, with tourists preferably choosing destinations with a low number of tourists and good sanitary conditions. A preference for places with outdoor activities or nature-related are characteristics looked for, as well as domestic destinations within the residency country. International travel has in destinations with a low number of COVID cases an attraction factor also (Santos et al., 2020).

Recovering from COVID-19 has become a tourism research urgency, and the importance of resilience is clear to help build a quick and effective response and is a significant part of the ongoing research (McCartney et al., 2021). Resilience is a concept that moves around “(…) between disciplines, between academia and public use, or between contexts,” and “it takes on slightly different meanings as it moves” (Rogers et al., 2020, p. 4). Resilience can be seen as a capacity to resist being “put down,” but also as the ability to recover and thrive from traumatic situations (Harms et al., 2018). As a personal trait, resilience may allow people to manage negative situations better (Liu et al., 2020). Research is showing that resilience has a mediator effect between personality traits and subjective wellbeing and stress experienced at the start of the pandemic, which leads to considering resilience as a protective factor to an adaptive reaction in the face of stressful experiences (Kocjan et al., 2021). Expanding this concept, “The resilience level of how the community responds to the disruption caused by the lockdown and stress caused could influence city resilience” (McCartney et al., 2021, p. 7). Research in resilience must go beyond how to come out of a crisis and develop future resilience (McCartney et al., 2021). Tourism must increase its resilience. This can be made by diversification: develop new business models; improve sustainability and digitalization (Santos et al., 2020). As scholars are acknowledging, “From now on, the bet should not be on the increase in visitor numbers but on ‘better, more comfortable travel, personalized service, while maintaining affordable prices”’ (Abbas et al., 2021, p. 6).

The changes in tourists’ behaviors with the pandemic allow an opportunity for research and innovation in tourism. A preference for safe and healthy environments is expected. Also, a shift from overtourism destinations to less busy ones, emphasizing rural and nature tourism, is already being seen. This can be an opportunity to help places that are more remote to flourish, and at the same time diminish the effects of overtourism in others, as seen before the pandemic (Santos et al., 2020). But also, the changes in tourists’ preferences can be opportunities to achieve the Sustainable Development Goals (Santos et al., 2020), including the goal for health and wellbeing. As acknowledged by scholars “(…) in wellbeing, it is possible to have multiple directions and starting points. It, however, makes sense that research should be venturing toward new grounds and eudaimonic wellbeing seems a natural approach as it is a concept related to the idea of personal fulfillment and development that people are looking for” (Garcês et al., 2020, p. 113).

Thus, for the current research wellbeing and resilience were chosen as the main variables. Wellbeing was chosen because, beyond being a European Sustainable Goal for 2030, it is also a rising motivator for traveling and tourism. Resilience was chosen because it is a concept intimately related to wellbeing, that helps to deal effectively with adversity. Beyond that, it is part of many countries’ strategic planning to deal with the pandemic. So, considering the importance of wellbeing for tourism and the need for resilience for all actors of this industry, this study tries to answer the question of how are wellbeing and resilience being used in tourism as positive strategies to deal with the pandemic negative consequences? Particularly, this research aims to analyze early positive approaches and attitudes to respond to COVID-19 negative impact on tourism everyday activities that have at its core wellbeing and resilience, two main variables of the Positive Psychology field of studies, which is the theoretical framework that guides this current research. Tourism everyday activities in the current study were considered people’s (tourists, residents, workers/stakeholders) actions and behaviors in activities linked to tourism.

## Methods

A systematic literature review was conducted using the Preferred Reporting Items for Systematic Reviews and Meta-Analyses – PRISMA (Moher et al., 2009). Considering the study aim, the following search terms were chosen Wellbeing; Resilience; Tourism; and Pandemic. The research took place in January 2021 using the Online Knowledge Library (B-On) and all the available databases on this platform which include: Complementary Index, SCOPUS, Academic Search Complete; Science Citation Index, Business Source Complete; MEDLINE, Supplemental Index, ScienceDirect; Directory of Open Access Journals, Social Sciences Citation Index, IEEE Xplore Digital Library, arXiv, Gale in Context: Science; Library, Information Science & Technology Abstracts, Arts and Humanities Citation Index, Gale Literature Resource Center, ERIC, SciELO, SciTech Connect, RCAAP, Dialnet, Government Printing Office Catalog, University Press Scholarship Online, Research Starters, Digital Access to Scholarship at Harvard (DASH), UC Digitalis; Oxford Scholarship online; SSOAR – Social Science Open Access Repository; eBook Index, Oxford Handbooks Online; and OAPEN Library. The search focused on scientific articles published between 2020 and 2021 in the English language.

The inclusion criteria used were (a) scientific articles published between 2020 and 2021; (b) articles written in English; (c) articles with the search terms included in its keywords; (d) scientific research articles with peer review; and (e) articles mainly focused on the search terms. The exclusion criteria used were (a) scientific articles published before 2020; (b) articles not written in English; (c) articles that did not include the search terms; (d) articles not peer-reviewed; and (e) articles not mainly focused on the search terms. The search was focused on the article’s keywords since this represents the core concepts of the articles.

The first search done on B-On crossed “Wellbeing or wellbeing or well-being” AND “Tour?sm*” AND “Pandemic or COVID-19 or coronavirus.” The second search was also done on B-On crossed the search terms: “Resilient?e” AND “Tour?sm*” AND “Pandemic or COVID-19 or coronavirus.” The Boolean operator “AND” was used to ensure that all three terms were included in the search and “OR” to ensure all variations for the terms “wellbeing” and those related to the “Pandemic.” The truncation symbol “*” was used to guarantee the inclusion of words with the same origin, and the “?” to include singular and plural forms. Inclusion/exclusion criteria (a); (b), (c), and (d) were applied through the online features of B-On, and criteria (e) was done manually.

Research led to the identification of 32 records in the following databases: Directory of Open Access Journals, Social Sciences Citation Index, ScienceDirect, Supplemental Index, Complementary Index, and SCOPUS. Four duplicates were found and removed, leading to 28 articles. Further analysis led to the exclusion of 11 more articles with criteria violations, namely, six commentaries; one editorial; and four records not mainly focused on the search terms. These criteria violations were encountered after applying inclusion/exclusion criteria (e) through a qualitative screening of each article abstract and/or full-text. Thus, in the end, a total of 17 scientific articles were considered as meeting all inclusion criteria, and hence, were further analyzed.

## Results

**FIGURE 1:**
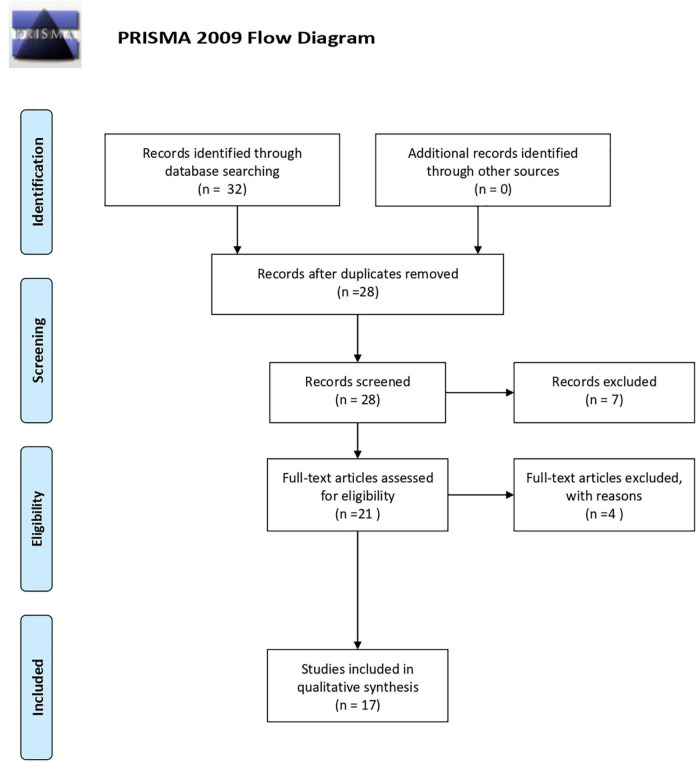
Flow diagram following PRISMA (2009) guidelines.

From the systematic literature review, only 32 records were first found. The application of the inclusion and exclusion criteria led to a total of 17 studies to be included in the final sample. The PRISMA (Moher et al., 2009) flow diagram for this research can be seen in Figure 1.

Despite the small number of articles, it is an indicator of interest by researchers on the importance of thinking about wellbeing and resilience amid the COVID-19 pandemic. Despite having restricted the search to 2020 and 2021, this decision was made solemnly with the intend to analyze the most current research regarding the use of positive variables such as wellbeing and resilience during the pandemic, which was only acknowledged by the World Health Organization (WHO) as a pandemic in March 2020 (World Health Organization [WHO], 2020). Table 1 presents the number of articles published in 2020 and January 2021 (data collection retrieval month).

**TABLE 1:** Number of articles distributed between 2020 and 2021.

Search terms	2020	2021
Wellbeing and tourism and pandemic	3	1
Resilience and tourism and pandemic	8	5

From Table 1, it is possible to acknowledge a low number of published articles related to the search terms on the selected dates. However, despite the low number of published articles, this research was done at the end of January 2021, almost a year after COVID-19 has been declared a pandemic (World Health Organization [WHO], 2020). In this short time gap, the existence of already published materials at this time highlights the current need to learn and explore more the impact of the pandemic and how to restart tourism’s everyday activities with a positive outlook.

From the analysis of Table 2, a clear emphasis is made on the resilience concept, with three times more articles on this topic than on wellbeing. This leads to thinking resilience as a positive psychological construct that reflects the skills to deal with adversity and is seen as important and as a positive asset and attitude to ensure the survival and future thriving of tourism.

**TABLE 2:** Number of articles distributed according to the positive variables: Wellbeing and resilience.

	Wellbeing	Resilience
Tourism and pandemic	4	13

In Table 3, all assessed articles’ main findings are summarized.

**TABLE 3:** Articles’ main findings.

Author(s)	Methodology	Target group	Main conclusion (s)
Agrusa et al., 2021	Survey through questionnaire *N* = 371	-Tourists	-COVID testing should be a travel requirement (before travel and on arrival) -COVID can be an opportunity to reset tourism goals and include residents in sustainable product development
Yang et al., 2020	Online survey *N* = 370	-Tourists	-Discrimination of tourists from COVID affected areas led to anxiety and depression symptoms, ruminations, including loss of wellbeing upon return home
Chua et al., 2020	Self-reported questionnaire	-Tourists	-Negative affected influenced perceived health risk and induced mental wellbeing and perceived uncertainty -Mental wellbeing predicted attitudes toward international travel and temporal avoidance behavior -Perceived uncertainty predicted short-term avoidance behavior
Buckley and Westway, 2020	Internet-based ethnography (netnography)	-Tourists -Enterprises	-Commercial outdoor tourism enterprises can contribute to tourists’ wellbeing and mental health following COVID negative effects
Sobaih et al., 2021	Questionnaire survey	-Enterprises	-Restaurant owner-managers expressed more resilience than hotels counterparts -Resilience had a direct and positive impact on sustainable tourism development and indirect influence through performance
Prayag, 2020	Review	-Organizations, destination, and tourists	-Resilience is an important future research area -COVID as opportunity to reset the whole tourism industry
Bhaskara and Filimonau, 2021	Qualitative research – qualitative paradigm	- Businesses	-Importance of disaster planning/management, and organizational learning, to enhance disaster resilience of tourism businesses not only for COVID-19 recovery, but for future crisis
Sharma et al., 2021	Systematic review	- Tourism as a whole	-A new resilience-based framework for tourism focused on: government response, technology innovation, local belongingness and consumer/employee confidence
Zheng et al., 2021	Quantitative research	- Tourists	-Threat severity and susceptibility can lead to travel fear -Travel fear can lead to different coping strategies and increase resilience and adoption of cautious travel behaviors
Sigala, 2020	Critical review	-Tourism as a whole	-COVID as a transformational opportunity
Setthachotsombut and Sua-iam, 2020	Mixed method, online questionnaire, and interviews	- Entrepreneurs	- Resilience has a positive impact on business performance
Vo-Thanh et al., 2020	Mixed-method, quantitative data through questionnaire and semi-structure interviews	-Hotel employees	-Satisfied employees with the organization COVID-19 responses positively influences job performance -Employees satisfaction help to maintain their wellbeing, leading them to adopt positive behaviors/attitudes to maintain their job performance
Filimonau et al., 2020	Quantitative survey	-Businesses	-Organizational resilience, including their COVID-19 response, and corporate social responsibility practices affect perceived job security and influences organizational commitment
Khan et al., 2020	Econometric approach	-Businesses	-Museums and historical places, performing arts, and sports show low resilience -Accommodation initially showed high vulnerability, but also signs of high resilience
Alonso et al., 2020	Qualitative research with purposeful sampling method	-Businesses	-Need to change and adjust to deal with COVID-19, including creating new revenue streams and new post-COVID-19 operational regime
Wen et al., 2020	Critical Review	-Tourists	-The pandemic will affect tourism/tourists behaviors -Increase interest in free and independent travel, including health and wellness tourism, slow and smart tourism
Mao et al., 2020	Questionnaire survey	-Businesses	-Corporate social responsibility positively impacts employee self-efficacy, hope, resilience and optimism trough employee satisfaction with COVID-19 responses

From an in-depth analysis of the sample findings, it is possible to see two major tourism actors of interest: tourists and businesses. Regarding tourists, there is a wide range of topics studied. Some examined tourists’ perceptions about wellbeing, highlighting the importance of safety measures before travel or even upon arrival, such as getting tested for COVID-19 (Agrusa et al., 2021). Others (Yang et al., 2020) emphasized the need to revisit travel-induced wellbeing, and the need to rethink it, particularly in the long term, since some tourism experiences such as the ones that occurred during the COVID-19 pandemic, brought a loss of wellbeing after the trip, particularly perceived discrimination, thus questioning the literature that considers tourism as an induce-wellbeing activity (Yang et al., 2020). Wellbeing was also considered as an important variable in predicting attitudes toward international travel and temporal avoidance behaviors (Chua et al., 2020). Another research (Zheng et al., 2021) studying different psychological variables and travel behavior in Chinese tourists, highlighted the positive benefits of “(…) psychological resilience on individuals’ intention to adapt caution travel after the pandemic outbreak.” Other researchers (Wen et al., 2020) predicting how COVID-19 will affect tourists’ behaviors emphasized a growing interest in health and wellness tourism, among others. Another one of the studies (Buckley and Westway, 2020) also acknowledged the psychological positive effects of walking-in-nature tourism, emphasizing an entrepreneur opportunity to promote wellbeing, particularly when thinking about recovering from the lockdowns that COVID-19 brought to the world.

As highlighted before, businesses were a focus of interest that stand-out in the current systematic research. In this regard, the importance of resilience to sustainable tourism development amid the pandemic is clear (Sobaih et al., 2021). Also, the importance of organizational learning and business preparedness to deal with crisis and disasters is emphasized, which can lead to business resilience to overcome the negative impacts of such disasters, not only COVID-19, but future ones too (Bhaskara and Filimonau, 2021). The positive impact of resilience on business performance is also highlighted in another study findings (Setthachotsombut and Sua-iam, 2020). The need for businesses to implement actions and changes to cope with the pandemic and its impact is clear (Alonso et al., 2020). Some studies in this regard highlight that satisfied employees with the organization COVID-19 responses positively influence job performance. Employees’ satisfaction may help to maintain their wellbeing, and therefore, they reciprocate through positive behaviors/attitudes (Vo-Thanh et al., 2020). Again, the importance of corporate social responsibility to maintain not only employee’s resilience but also other positive psychological variables such as self-efficacy, hope, and optimism is empathized (Mao et al., 2020). The importance of organizational resilience to organizational commitment, and the fact that resilience influences “the scope of adoption of anti-COVID-19 measures” is again emphasized (Filimonau et al., 2020). Overall, another research stated that different sectors of the leisure and hospitality industry showed different resilience “levels” and some signs of recovery, still the pandemic is a hard situation and will endure a long-run recovery period (Khan et al., 2020).

While tourists and businesses have a clear interest in research, some studies also highlight tourism as a whole unit, acknowledging the importance of resilience of destinations, enterprises, and tourists and its study, but also to see COVID-19 as an opportunity to reset tourism (Prayag, 2020). A resilience framework for the tourism industry, highlighting this positive variable importance is acknowledged, and that smaller enterprises can also gain and ensure wellbeing at a bigger scale, while also promoting a more sustainable tourism (Sharma et al., 2021). Research about COVID-19 can be a way to innovate tourism having sustainability and wellbeing as centerpieces (Sigala, 2020).

In this analysis, it is also an important topic to rethink the future of tourism in the post-COVID era (Agrusa et al., 2021). COVID-19 can be an opportunity to rethink tourism policies and strategies to ensure stability between the wellbeing of residents, tourists, and products, particularly in areas where overtourism was already a big issue among residents (Agrusa et al., 2021).

## Discussion

Overall, the current systematic literature review highlighted the importance of wellbeing and resilience in tourism’s everyday activities during the COVID-19 pandemic. Tourism is one of the industries worldwide, that was most affected (if not the most affected) by the pandemic with a reduction of international arrivals from January 2020 to 2021 of more than 80% (UNWTO, 2021c). This number is astonishing and something that has never occurred before such a scale.

The pandemic is a threat to most activities in tourism and in many other sectors, and for all Humankind. Particularly it puts at risk the achievement of 2030 Sustainable Development Goals, including the mental health and wellbeing of all people (Passavanti et al., 2021). Tourism was (and still is) tremendously affected by COVID-19, with a drop of more than 64% in receipts (UNWTO, 2021b), thus, affecting destinations, locals, and tourists. Resilience has come as a major goal and key process to overcome the challenges imposed by the pandemic. A big emphasis of this concept is seen on tourism stakeholders who are trying to survive COVID-19 impacts (McCartney et al., 2021). The urgency to promote and increase resilience can be seen in the number of articles that focused on resilience comparatively to wellbeing in the current systematic literature review. The uncertainty that the pandemic brought made it urgent for the tourism industry to find new ways to overcome the difficulties. This can be observed in the results, where topics related to how to deal with the current crisis and even future ones (Alonso et al., 2020; Bhaskara and Filimonau, 2021) or the benefits of resilience for businesses (Setthachotsombut and Sua-iam, 2020) are highlighted and accentuated by the research. Resilience plans should be standard practice for all tourism stakeholders. While a pandemic was not something foreseen, it should be thought of as a warning for better planning and management in case of disasters or crises in tourism. Being an economic activity, tourism, highly dependent on external factors, such as weather, social crisis, or security, going forward, destination’s policymakers must prevent future crisis setbacks by planning and ensuring resilience to deal with whatever the next threat may be. In practical terms, such planning should be considered in national and local policies, but also as an internal business policy. In here, the introduction of, for example, policies to work remotely can be identified to ensure a smooth transition from on-site to on-line or even hybrid performances.

Another important result from this systematic analysis is the fact that research in wellbeing and resilience has mainly been focused on tourists and above all on businesses. This situation is also acknowledged by the literature (Vada et al., 2020) where research has mostly been centered on tourists leaving behind local communities and tourism workers. Businesses have a particular interest in how to face COVID-19 and be resilient to ensure the thriving of the industry. Although in this review tourism workers were acknowledged in some articles (Mao et al., 2020; Vo-Thanh et al., 2020), more needs to be done because their wellbeing is also affected by the pandemic (McCartney et al., 2021) and they are one of the key pillars of tourism (Garcês et al., 2020). Thus, businesses should invest more in wellbeing of their workers since they are the forefront of tourism businesses but also its background. A worker that feels safe and secure will deliver a more satisfactory service that will in return improve the tourists’ satisfaction. However, the complete lack of research on local communities and their residents is of concern, because the pandemic has affected global livelihoods and destinations have no longer tourists (Abbas et al., 2021), which will have impacts on places that have tourism as the main economy. Therefore, studies about locals’ wellbeing and how they face pandemic’s incoming repercussions should be developed, which will hopefully lead to the development of strategies to help the residents of tourism destinations deal with this crisis aftermath while promoting their wellbeing and mental health.

Another interesting result is the disparity between the articles that focused on wellbeing and resilience. Resilience shows a far greater interest. However, it is important to not forget that tourism can be a way to experience wellbeing (Garcês et al., 2020), and although the number of articles focused on wellbeing is much less, they show that this variable should not be forgotten, highlighting the fact that COVID-19 will affect the interest of tourists, moving them to destinations that have in attention wellness and wellbeing endeavors (Wen et al., 2020). Policymakers and stakeholders have in here a “gold” opportunity to innovate. Wellbeing and wellness can be attractive factors for new tourists and thus open doors to developing new products and activities in destinations. These changes in tourists’ behaviors should be seen as opportunities to “refresh” tourism and even to solve pre-COVID problems in some destinations such as overtourism. Tourists will be now looking more for quieter places, with outdoor experiences and in nature (Santos et al., 2020). This is interesting because research in pre-COVID times has linked nature to feelings of wellbeing (Garcês et al., 2018), and results in the current study already show this trend highlighting the psychological benefits of walking in nature, for example (Buckley and Westway, 2020). Thus, stakeholders can have in wellness and nature products a source to innovate and promote tourists’ wellbeing.

It is also important to note that a small number of articles in this systematic review highlight the future of tourism after COVID-19. This emphasis gives hope for a positive outlook for the future, focusing on the proactive and preventive measures that will help the survival and thriving of the industry, highlighting the importance of developing new policies and strategies to promote wellbeing among all tourism actors (Agrusa et al., 2021). This idea is in accordance with the literature that acknowledges the need to focus on the quality and personalization of services at reasonable prices (Abbas et al., 2021), build new business models, and enhance sustainability and digitalization (Santos et al., 2020) on the post-COVID era. But to become a more resilient industry and a promoter of wellbeing, the industry needs to first think of what has gone wrong, identify the stressors, and develop contingency plans and strategic ones to deal with present and future uncertainty, including crisis. Additionally, it is relevant to notice that the new emerging field of existential positive psychology focus the importance of suffering to flourishing and highlights that dealing with negative situations will allow its transformation to a sense of accomplishment and mature happiness (Wong, 2021). In this new emerging trend, wellbeing is simultaneously a process and an outcome in dealing with the search for positive life goals and transforming the negative situations into better ones (Wong, 2020). Thus, whilst the pandemic has had so far, a tremendous negative impact in tourism, it can also be seen as an opportunity to innovation, and to build the sector resilience while promoting wellbeing for the destinations, the stakeholders, and the tourists. With this new vision of existential Positive Psychology, it is possible to reflect that while COVID-19 brought with it a lot of suffering including for tourism stakeholders, it is also an opportunity to learn from and develop new strengths and simultaneously to improve people tourism experiences.

This study thus highlights an interest in resilience and wellbeing in tourism. However, there is still space for growth. Destinations’ policymakers and all involved in tourism must prepare better for a future crisis, with resilience programs that consider strategies to surpass the adverse outcomes of such crisis, not only for businesses but also for their workers and their residents. At the same time, developing innovation through wellbeing can be a differentiator factor for the destinations, but it can also help promote tourists, locals, and stakeholders’ mental health. New products with a focus on wellness, nature, or rural places can be starting points. This research also brings with it the importance of (re)thinking tourism not only in economic gains, but also in psychological aspects that can have great impact on the restart of the tourism during this pandemic and beyond. Also, this study was developed considering only the COVID-19 pandemic, and what has been done during this situation in terms of wellbeing and resilience in tourism. Thus, it shows the impact of COVID-19 pandemic, a current world problem, in tourism studies. Additionally, world institutions such as OMS and governments of many countries have highlighted the need to be resilient and promote wellbeing in the face of this health crisis, and the tourism industry, as one of the most affected, is no exception.

As for the study limitations, this research was done in January 2021, thus throughout the year, there is an expectation that more studies about the impact of COVID-19 in tourism, particularly considering the study variables will have been published. Thus, it will be important to further study these variables in future studies. The use of keywords may have limited the scope of the research. Therefore, in future research, expanding the search to the title and abstract may allow for the generation of a more significant number of articles on these topics. Also, it will be important to develop practical interventions on how wellbeing and resilience can be key points in tourism. Thus, not only tourists or businesses should be the focus, but also the locals and the workers of this sector.

Overall, in this systematic literature review, it was possible to see a clear focus on the impact and recovery of businesses from COVID-19 pandemic, with resilience as an important variable to achieve it. It is the authors’ belief that this article can contribute particularly to those countries and regions that exclusively depend on tourism everyday activities and that were severely affected by the pandemic, since many depend entirely on the tourism industry to economically survive. This article also hopes to contribute with some ideas and suggestions of how to introduce wellbeing and resilience in the tourism industry, allowing for potential course of action to be taken by all those involved in it. Concluding, while the articles that met all the inclusion criteria were few, this systematic review highlights the concerns of the sector and the urgency to rebound quickly and effectively, and restart tourism and its everyday activities safely and with a positive attitude.

## Data Availability Statement

The raw data supporting the conclusions of this article will be made available by the authors upon request, without undue reservation.

## Author Contributions

All authors listed have made a substantial, direct, and intellectual contribution to the work, and approved it for publication.

## Conflict of Interest

The authors declare that the research was conducted in the absence of any commercial or financial relationships that could be construed as a potential conflict of interest.

## Publisher’s Note

All claims expressed in this article are solely those of the authors and do not necessarily represent those of their affiliated organizations, or those of the publisher, the editors and the reviewers. Any product that may be evaluated in this article, or claim that may be made by its manufacturer, is not guaranteed or endorsed by the publisher.
